# Bidirectional Glenn shunt with tricuspid valve resection in patients with infective endocarditis

**DOI:** 10.1016/j.xjtc.2022.03.002

**Published:** 2022-04-02

**Authors:** Yi-Chia Wang, Yih-Sharng Chen, Nai-Hsin Chi, Shu-Chien Huang

**Affiliations:** a Department of Anesthesiology, National Taiwan University Hospital, Taipei, Taiwan; b Department of Surgery, National Taiwan University Hospital, Taipei, Taiwan

**Figure:**
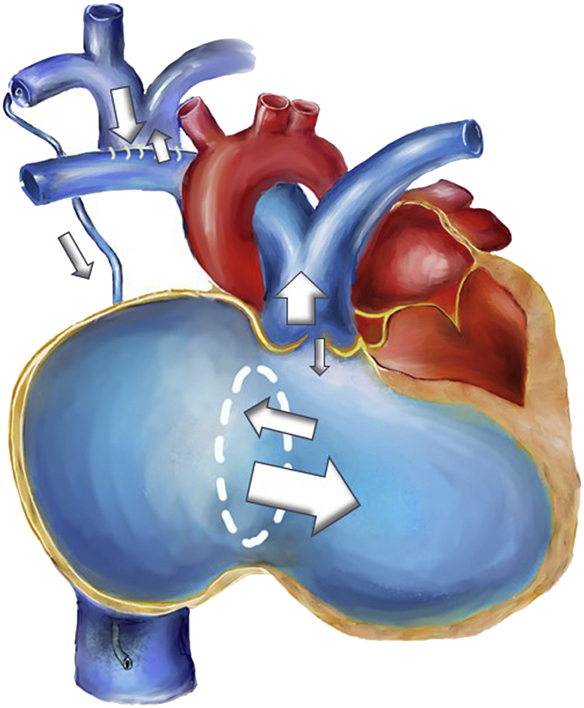
RV dysfunction after BDG and TV resection.

**Central Message**

BDG unloads the RV volume in acute endocarditis patients after TV resection, but severe tricuspid regurgitation leads to RV dysfunction in the long term.

In drug abusers with infective endocarditis, tricuspid valve (TV) replacement involves high recurrence risk.^1^ We have treated these patients with resection of infected tricuspid valve leaflets, TV annular reduction, and bidirectional Glenn shunt (BDG).^2^ BDG diverts flow from the superior vena cava (SVC) to pulmonary artery to unload right ventricular (RV). In this study, we report the follow-up of patients with TV endocarditis who received BDG with TV resection.

## Clinical Data

We retrospectively reviewed the medical records of 4 patients who received BDG and TV resection.^2^ The study was approved by the Ethical Committee of National Taiwan University Hospital (no. 202108103RINA; approval date September 24, 2021). Informed consent was waived because of deidentified patient data. Three patients successfully overcame their drug addiction in 1 year, and 1 patient was lost to follow-up because of criminal problems (Table 1). Their functional status improved. Moreover, patient 1 delivered a healthy baby 5 years after surgery. However, congestive heart failure progressed after delivery. Because her right atrium (RA) and RV were dilated (right ventricular end-diastolic volume index = 117.7 mL/m^2^), and RV systolic function deteriorated (right ventricular ejection fraction = 39%), her TV was replaced with a 31-mm porcine valve (Hancock II; Medtronic). After TV replacement and RA closure, the SVC was removed from the pulmonary artery and reanastomosed to the RA through continuous suture. She had sinus rhythm afterwards. Her RA and RV regained their normal sizes 2 years after surgery, with improved functional status.

**Table 1:** Clinical course of infective endocarditis after initial treatment with BDG and TV resection

No.	After first operation (BDG + TV resection)			Second operation (SVC reconstruction + TVR)									Outcome					
	Fc status	SVC/RA pressure, mm Hg	Rhythm	Year after BDG	Fc status	SVC/RA Pressure, mm Hg	Rhythm	Echo/MRI					Follow-up period	Rhythm	Echo			
								RA	RV	RVOT antegrade flow	Tricuspid valve	LV						
															RA	RV	TR	LV
1	1	18/12	Sinus	7	3	18/12	Sinus tachycardia RBBB	Dilate	Dilate RVEDVI = 117.7 mL/m^2^ RVEF:39%	Mild PR	Moderate-to-severe TR	LVEF 59% diastolic dysfunction	9	Sinus	Normal	Normal	Moderate PG: 26.1 mm Hg	Fair systolic and diastolic function
2	1	20/10	Sinus	7	3	16/10	Sinus tachycardia RBBB	Dilate	Dilate RVEDVI = 100.96 mL/m^2^ RVEF: 58%	Mild PR	Severe TR Mild TS	Mild LV dilatation Fair systolic function diastolic dysfunction	6	Sinus	Normal	Normal	Mild PG: 24 mm Hg	Fair systolic and diastolic function
3	1	16/9	Sinus	16	3	13/8	Sinus tachycardia RBBB	Dilate	Dilate RVDd: 3.9 cm RVFAC: 25%	Severe PR, decreased antegrade flow	Severe TR	Small LV Diastolic dysfunction	0.6	Sinus	Dilate	Dilate	Mild PG: 17.8 mm Hg	Fair systolic and diastolic function

*BDG*, Bidirectional Glenn shunt; *TV*, tricuspid valve; *SVC*, superior vena cava; *TVR*, tricuspid valve replacement; *Fc status*, New York Heart Association functional status; *RA*, right atrium; *MRI*, magnetic resonance imaging; *RV*, right ventricle; *RVOT*, right ventricular outflow tract; *LV*, left ventricle; *TR*, tricuspid regurgitation; *RBBB*, right bundle branch block; *RVEDVI*, right ventricular end-diastolic volume index; *RVEF*, right ventricular ejection fraction; *PR*, pulmonary regurgitation; *LVEF*, left ventricular ejection fraction; *PG*, pressure gradient; *TS*, tricuspid stenosis; *RVDd*, right ventricle diastolic diameter; *RVFAC*, right ventricular functional area change.

Patient 2 experienced exertional dyspnea 4 years after BDG with TV resection. The maximal oxygen consumption was only 9.77 mL/min/kg (37% of the predicted value). Cardiac magnetic resonance imaging revealed severe RA dilatation, mild RV dilatation (right ventricular end-diastolic volume index = 100.96 mL/m^2^), and preserved RV contractility (right ventricular ejection fraction = 58%) (Video 1). SVC angiography revealed patent BDG with multiple collateral veins from the right subclavian vein to the inferior vena cava (IVC). She received TV replacement with a 33-mm St Jude Medical Epic porcine bioprosthetic valve, and her SVC was reconnected to the RA by using a 22-mm GORE-TEX graft 7 years after first operation. Her RV size normalized in the first year after surgery. She had sinus rhythm and improved exercise function during follow-up.

Patient 3 first experienced exertional dyspnea 2 years after BDG and TV resection. Echocardiography revealed patent BDG, severe RA dilation, RV dilatation (RV diastolic diameter = 3.9 cm), and left ventricle (LV) compression. Decreased antegrade flow in the right ventricular outflow tract was disclosed because of severe tricuspid regurgitation. Atrial fibrillation was also noted. The dyspnea exacerbated during follow-up; hence, her TV was replaced with a 33-mm SJM Masters Series Mechanical Heart Valve 16 years after the first operation. The SVC was re-anastomosed to the RA with continuous suture. She had sinus rhythm afterwards, and her LV compression was relieved, with improved LV diastolic function.

## Discussion

The long-term outcomes of BDG and TV resection in adult patients have limited reference. We previously demonstrated that BDG with TV resection provides a good short-term hemodynamic profile^2^ and an adequate observation period for infection control and drug abstinence. However, because of severe tricuspid regurgitation, RV unloading after BDG is insufficient in the long term. TV insufficiency causes blood backflow, which leads to progressive right-side chamber dilatation, function deterioration, and decreased right ventricular outflow tract antegrade flow^3^ (Video 1). TV replacement corrects this and improves functional status.

**Figure 1:**
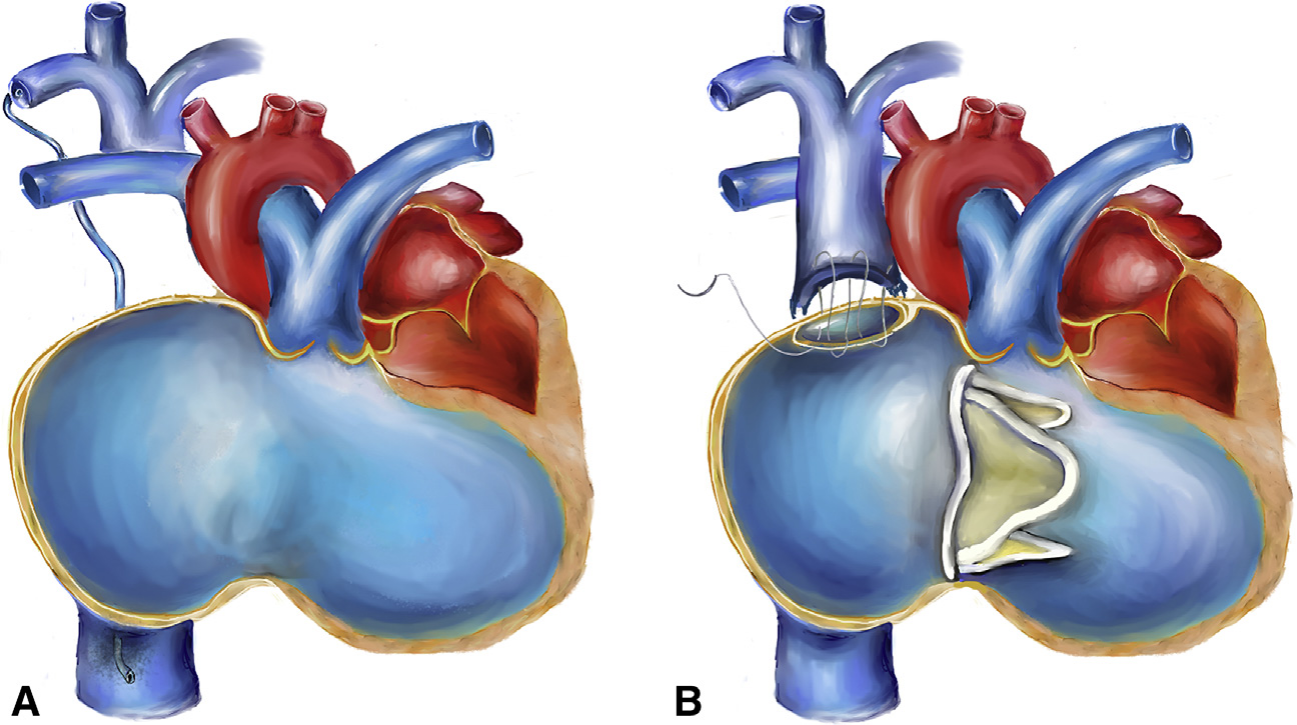
TV endocarditis repaired with (A) BDG and TV resection resulted in TR, collaterals from the subclavian vein to the IVC, decreased RVOT antegrade flow, RA and RV dilatation, and impaired systolic function with compromised LV diastolic function. (B) We took down BDG, reanastomosed the SVC to the RA, and replaced the TV. *TV*, Tricuspid valve; *BDG*, bidirectional Glenn shunt; *TR*, tricuspid regurgitation; *IVC*, inferior vena cava; *RVOT*, right ventricular outflow tract; *RA*, right atrium; *RV*, right ventricle; *LV*, left ventricle; *SVC*, superior vena cava.

BDG is applied in congenital heart disease with insufficient RV function.^4^ BDG failure has several risk factors, such as pulmonary hypertension and elevated left atrial pressure. There was no evidence of pulmonary emboli or pulmonary hypertension in our patients, but all our patients had LV diastolic dysfunction before TV replacement. One patient even had a reduced LV volume due to septal displacement by RV dilatation. Last but not least, the pressure difference in the SVC and IVC could lead to venous collateral circulation, as observed in one of our patients. If venous collateral circulation from the SVC to IVC is significant, the RV workload increases, and BDG lost its unloading function (Figure 1). Because tricuspid insufficiency disappears after TV replacement, RV unloading by using BDG is no longer needed. Thus, we converted one and a half circulation to the biventricular status through the reconnection of the SVC to the RA in all our patients.

In summary, TV resection with BDG can provide a stable hemodynamic condition in the short term and offer an adequate observation period for drug abstinence. In the long-term follow-up, prosthetic TV replacement and SVC reimplantation are needed in case of severe tricuspid insufficiency and worsened RV function.

## Supplementary Data

Video 1 Prominent TR, dilated RA and RV; patent Glenn shunt without junctional stenosis. Video available at: https://www.jtcvs.org/article/S2666-2507(22)00184-5/fulltext.
