# Supplementary figures and images for: Bidirectional Glenn shunt with tricuspid valve resection in patients with infective endocarditis
Source: JTCVS Tech. 2022 Apr 2;13:58–61. doi: 10.1016/j.xjtc.2022.03.002 (PMC9196930; doi:10.1016/j.xjtc.2022.03.002)

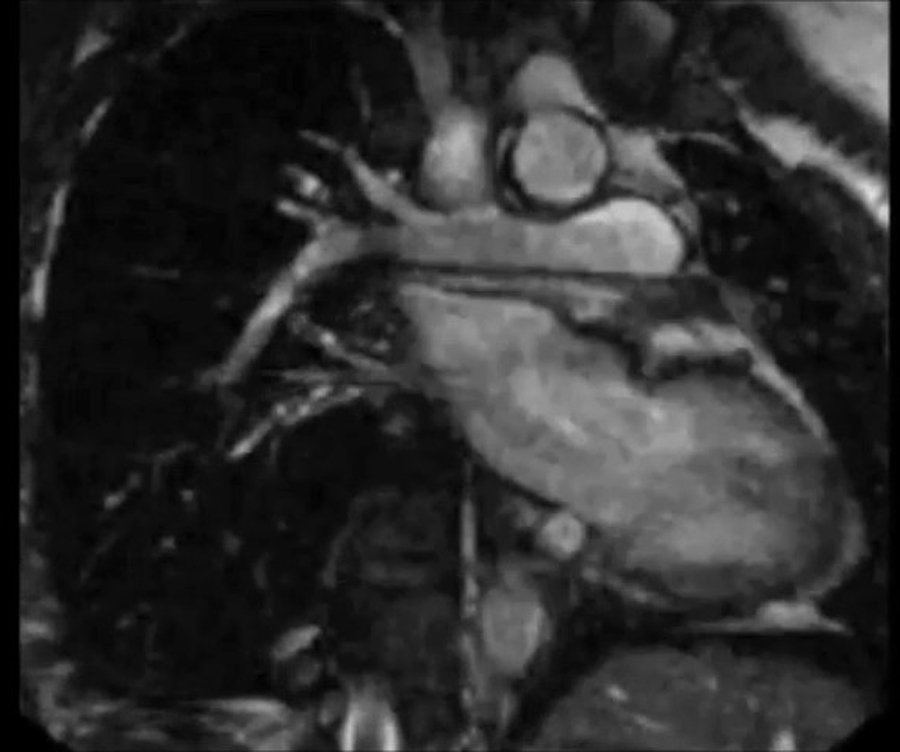

Supplement: Video 1 — Prominent TR, dilated RA and RV; patent Glenn shunt without junctional stenosis. Video available at: https://www.jtcvs.org/article/S2666-2507(22)00184-5/fulltext. [file fx2.jpg]
